# Gender differences of clinical and polysomnographic findings with obstructive sleep apnea syndrome

**DOI:** 10.1038/s41598-021-85558-y

**Published:** 2021-03-15

**Authors:** Xiaobo Zhou, Bo Zhou, Zhe Li, Qiao Lu, Shaoping Li, Zhongyin Pu, Fang Luo

**Affiliations:** 1 Department of Psychosomatics, Sichuan Academy of Medical Sciences & Sichuan Provincial People’s Hospital, Chengdu, China; 2 Department of Psychiatry, Panzhihua Shengtai Rehabilitation Hospital, Panzhihua, 617000 China

**Keywords:** Psychiatric disorders, Risk factors

## Abstract

Obstructive sleep apnea syndrome (OSAS) is underdiagnosed in females and gender differences in clinical and polysomnographic findings have not been widely investigated in China. We examined clinical and polysomnographic differences between males and females with OSAS in order to determine the influence of gender on clinical presentation and polysomnographic features. Data were collected from 303 adult patients diagnosed with OSAS (237 males and 66 females) from 2017 to 2019. All the patients completed physical examination, Epworth sleepiness scale, and whole night polysomnography. AVONA, univariate and multivariate logistic regression analyses were conducted to assess gender differences of clinical and polysomnographic findings with OSAS. *P* < 0.05 was statistically significant. The average age was 48.4 ± 12.6 years for females and 43.4 ± 12.4 years for males. Compared with female patients with OSAS, male patients were taller and heavier, had higher systolic blood pressure in the morning, shorter duration of slow wave sleep, more micro-arousal events, greater AHI, and more complex sleep apnea events. There are obvious gender differences of clinical and polysomnographic characteristics with OSAS. Understanding gender differences will contribute to better clinical recognition of OSAS in females as well as the provision of proper health care and therapeutic practice.

## Introduction

Obstructive sleep apnea syndrome (OSAS) is a common sleep disorder characterized by repetitive episodes of partial or complete upper airway obstruction, resulting in sleep fragmentation and cardiovascular comorbidity^1^. OSAS was thought to be very rare in females and a great number of epidemiological studies of OSAS included only males^2,3^. According to studies in the past, the male to female prevalence ratio of OSAS varied from 2:1–4:1 in the community to 8:1 or greater in the clinics^4–6^. It has been estimated that females with OSAS was under-diagnosed to a large extent^7^.

Inadequate understanding of sex difference in OSAS might be the root of the gender bias observed in OSAS. The National Sleep Disorders Research Plan^8^ acknowledged that there was a lack of inclusion of women in the past studies and has given priority to the exploration of gender differences in sleep disorders. Understanding gender differences in OSAS has currently been an important issue.

Gender differences in clinical and polysomnographic (PSG) findings have not been widely investigated outside North America^9^, and Chinese patients with OSAS may have different clinical features from North American patients. Therefore, the present study was undertaken to examine the gender differences in clinical and PSG findings in a subset of Chinese adult patients with OSAS and compare the findings with data from the literature.

## Results

A total of 303 adult patients who were diagnosed with OSAS based on PSG were the focus of the study. The male to female ratio was 3.59:1 with respect to 237 males and 66 females. The mean age was 43.4 ± 12.4 years for males and 48.4 ± 12.6 years for females. 24.2% of females and 7.5% of males were older than 60 years (Table 1).

**Table 1:** The age distribution of all the patients in genders.

	Female (n = 66)	Male (n = 237)	Statistics	*P*
Young age (18–44)	24 (36.4)	129 (54.4)		
Middle age (45–59)	26 (39.4)	90 (29.7)		
Young old age (60–74)	16 (24.2)	15 (6.3)		
Old age (> 75)	0	3 (1.2)		
Mean (SD)	48.4 (12.6)	43.4(12.4)	F = 8.360	0.004**

**P < 0.05.

Regarding body mass index (BMI) distribution, in the normal weight group, females were proportionally more (57.6 vs 40.9%), whereas in the overweight group, males were proportionally more (45.9 vs 28.7%). The proportion of obese patients was similar in male and female groups (11.4% vs 10.7%) (Table 2).

**Table 2:** BMI distribution of all the patients in genders.

BMI (kg/m^2^)	Female (n = 66)	Male (n = 237)	Statistics	*P*
< 18.5 Underweight	2 (3.0)	3 (1.3)		
18.5–25 Normal weight	38 (57.6)	97 (40.9)		
25–30 Overweight	19 (28.7)	109 (45.9)		
30–40 Obesity	7 (10.7)	27 (11.4)		
> 40 Pathological obesity	0 (0)	1 (0.5)		
Mean (SD)	24.6 (3.7)	25.9 (3.5)	F = 7.420	0.007**

**P < 0.05.

According to univariate and multivariate logistic regression analyses (Tables 3 and 4), compared with female patients with OSAS, male patients were taller (157.5 ± 5.0 cm, versus 169.8 ± 6.0 cm, respectively) and heavier (61.1 ± 9.6 kg versus 74.9 ± 11.5 kg, respectively). Male patients had higher systolic blood pressure in the morning (119.4 ± 13.6 mmHg versus 124.3 ± 16.1 mmHg, respectively), shorter duration of slow wave sleep (SWS) (52.7 ± 41.5 min versus 37.8 ± 27.3 min, respectively), more micro-arousal events (100.1 ± 77.0 versus 161.2 ± 125.5, respectively), more complex apnea events (2.7 ± 6.2 versus 18.2 ± 46.3, respectively). Apnea–hypopnea index (AHI) during total sleep time (TST) was greater in males than in females (24.3 ± 19.4 min versus 33.9 ± 25.5 min, respectively).

**Table 3:** Univariate logistic regression analysis of gender differences of clinical and polysomnographic findings with OSAS.

Variables	Female	Male	Statistics	*P*
**Demographic and clinical data**				
Age	48.4 (12.6)	43.4 (12.4)	F = 8.360	0.004**
ESS scoring	8.2 (5.6)	8.7 (5.9)	F = 0.481	0.488
Height (cm)	157.5(5.0)	169.8 (6.0)	F = 228.69	0.000**
Weight (kg)	61.1(9.6)	74.9 (11.5)	F = 79.01	0.000**
BMI	24.6 (3.7)	25.9(3.5)	F = 7.420	0.007**
**Blood pressure (mmHg)**				
SBP before sleep	119.7 (12.9)	125.1(15.5)	F = 6.546	0.011**
DBP before sleep	72.1 (9.9)	75.4 (11.9)	F = 4.305	0.039**
SBP in the next morning	119.4 (13.6)	124.3(16.1)	F = 4.834	0.029**
DBP in the next morning	73.4 (9.7)	77.5(11.8)	F = 6.189	0.013**
**Sleep parameters**				
**Sleep latency (min)**				
N1 sleep	14.1 (15.4)	9.4 (17.3)	F = 3.997	0.046**
N2 sleep	23.9 (29.9)	16.7(24.1)	F = 4.202	0.041**
N3 sleep	64.3 (76.8)	79.6 (92.6)	F = 1.525	0.218
REM sleep	146.8 (84.1)	131.5(77.4)	F = 1.942	0.165
TST (min)	451.7(69.8)	455.1(68.7)	F = 0.130	0.719
REM (min)	67.4(30.8)	71.9 (28.3)	F = 1.222	0.270
NREM (min)	384.2 (62.3)	397.6 (242.1)	F = 0.198	0.657
N1 + N2 (min)	331.5 (67.8)	359.8 (240.6)	F = 0.892	0.346
SWS (min)	52.7(41.5)	37.8(27.3)	F = 12.008	0.001**
Sleep efficiency	85.6 (10.5)	86.3 (12.1)	F = 0.174	0.677
**Respiratory parameters**				
Micro-arousals events	100.1 (77.0)	161.2(125.5)	14.157	0.000**
Arousals events	17.3(9.0)	18.5(11.6)	0.608	0.436
MAI	15.0(10.4)	24.5(17.0)	18.556	0.000**
AHI	24.3 (19.4)	33.9(25.5)	F = 7.970	0.005**
Mean SpO_2_	95.9 (2.0)	95.6 (1.7)	F = 0.853	0.356
Lowest SpO_2_	82.6 (8.8)	78.7 (14.1)	F = 4.476	0.035**
**Snoring**				
Frequency	179.1 (188.4)	20.2.1 (191.9)	F = 0.742	0.390
Duration (min)	70.(77.5)	67.5 (155.5)	F = 0.016	0.900
Percentage (%)	15.0 (16.4)	12.7 (13.9)	F = 1.293	0.256
**Obstructive apnea**				
Frequency	85.9(17.9)	131.4(186.2)	F = 7.221	0.008**
Maximum duration (min)	30.2(19.2)	41.3 (25.4)	F = 10.722	0.001**
**Centric apnea**				
Frequency	1.5(3.6)	4.1(8.9)	F = 5.284	0.022**
Maximum duration (min)	6.2(7.6)	11.5(10.5)	F = 14.508	0.000**
**Hypopnea**				
Frequency	115.7 (78.8)	105.5 (74.4)	F = 0.945	0.332
Maximum duration (min)	45.4 (14.1)	47.2 (12.2)	F = 1.120	0.290
**Complex sleep apnea**				
Frequency	2.7 (6.2)	18.2 (46.3)	F = 7.345	0.007**
Maximum duration (min)	12.1(15.9)	26.1 (23.6)	F = 20.353	0.000**
Awake time (min)	54.0 (52.4)	55.2 (58.5)	F = 0.022	0.882
Supine position (%)	63.8 (21.3)	52.3 (24.2)	F = 12.099	0.001**

*MAI* microarousal index, *AHI* apnea–hypopnea index, *SWS* slow wave sleep, *TST* Total sleep time, *SBP* systolic blood pressure, *DBP* diastolic blood pressure, *ESS* Epworth Sleepiness Scale.

**P < 0.05.

**Table 4:** Multivariate logistic regression analysis of gender differences of clinical and polysomnographic findings with OSAS.

Variables	B	Exp(B)	*P*
Height	0.364	1.438	0.000**
Weight	0.093	1.098	0.005**
SBP in the morning	0.040	1.041	0.030**
Duration of SWS	−0.024	0.976	0.003**
Frequency of micro-arousals	0.010	1.010	0.007**
AHI	−0.083	0.921	0.001**
Frequency of complex sleep apnea	0.090	1.095	0.047**
MAI			0.684
Frequency of centric sleep apnea			0.079
Frequency of obstructive apnea			0.853
Maximum duration of complex apnea			0.494
Maximum duration of centric apnea			0.604
Maximum duration of obstructive apnea			0.707
BMI			0.967
Age			0.931
SBP before sleep			0.527
DBP before sleep			0.627
DBP in the morning			0.878
N1 latency			0.393
N2 latency			0.811
Minimum S_P_O_2_			0.930
Supine position (%)			0.269

*MAI* microarousal index, *AHI* apnea–hypopnea index, *SWS* slow wave sleep, *TST* Total sleep time, *SBP* systolic blood pressure, *DBP* diastolic blood pressure.

**P < 0.05.

TST was similar in male and female patients (455.1 ± 68.7 min versus 451.7 ± 69.8 min, respectively). There were no gender differences in REM sleep stage, N_1_ and N_2_ sleep duration (67.4 ± 30.8 min versus 71.9 ± 28.3 min, respectively, 331.5 ± 67.8 min versus 359.8 ± 240.6 min, respectively). Sleep latency of N1 and N2 was similar in females and males (14.1 ± 15.4 min versus 9.4 ± 17.3 min, 23.9 ± 29.9 min versus 16.7 ± 24.1 min, respectively). There was no significant gender difference of sleep efficiency (85.6 ± 10.5% versus 86.3 ± 12.1%, respectively). Mean and minimum oxygen desaturation were similar between genders (95.6 ± 1.7% versus 95.9 ± 2.0%, 78.7 ± 14.1% versus 82.6 ± 8.8%).

## Discussion

The male to female ratio in our study was 3.59:1, which was consistent with previous studies^4–6^. According to previous literature, most of the female patients with OSAS were in menopause and older than male patients. Raluca et al pointed out that hormones especially progesterone might protect premenopausal women from developing OSAS and testosterone may be a risk factor of OSAS^10^.

Our study found that male patients were taller and heavier than female patients. According to the report on Nutrition and Chronic Disease Status of Chinese Residents (2015), the average height for males and females aged over 18 years old were 167.1cm and 155.8cm respectively, which was similar to our findings. Literature about the gender differences in BMI among OSAS patients were mixed. Most of the studies reported that females had higher BMI than males^11,12^ whereas other studies including our study showed that BMI could not play a role in the gender differences of OSAS patients^13^.

Our study showed that daytime sleepiness and snoring symptoms were similar between male and female patients with OSAS, and males showed more witnessed apneas. Andressa Silva et al.^14^ found that snoring and sleepiness were similarly common in females and males, but females went to see a doctor more often with a chief complaint of insomnia, which may partly explain the phenomenon of lower diagnostic rate of OSAS in females.

Our study showed that female patients had longer duration of SWS. Fewer studies have investigated gender differences in the SWS, and the data are more conflicting. Few studies indicated that there were no gender differences in the amount of SWS^15^, whereas other studies reported that males may have sustained reductions in SWS as compared with females over the lifespan^16–18^. It is well known that AHI is significantly decreased during SWS ^19^ and there is an attempt to explain it by higher upper-airway muscle activity and/or lower upper-airway collapsibility during SWS^20,21^.

Our study found that males have more severe OSAS than females, which was consistent with most of the previous studies^6,12,22–24^. The pathophysiologic mechanisms have not been well clarified. Differences in the dynamic properties of the upper airway may play a critical role. Elisa Perger et al.^25^ suggested that upper airways were stiffer in females than in males, so that females were less susceptible to collapse and males had greater pharyngeal collapsibility. In addition, female hormones may increase the upper airway dilator muscles tone, which helped to prevent airway collapse^26^.

Our study found that systolic blood pressure in the morning was significantly higher in males than in females. The micro-arousal events were also greater in males. Recently emerging evidence suggests that there is a causal link between OSAS and hypertension, and hypertension represents an independent risk factor of OSAS. In addition, the existence of micro-arousals is enough to cause an hypertension peak which might partly explain the results^27^.

Limitations of the study need to be pointed out. The patients enrolled in the study were from only one sleep disorder center, which might result in potential sampling bias. Larger, multi-center studies are required to explore the impact of gender on OSAS. A better understanding of gender differences of OSAS will be useful in better recognition, intervention and treatment of the syndrome in females.

## Methods

### Participants

A total of 303 adult patients with OSAS were referred to the sleep disorder center of department of psychosomatics, Sichuan Academy of Medical Sciences & Sichuan Provincial People's Hospital from 2017 to 2019. All patients completed physical examination, Epworth Sleepiness Scale (ESS) and PSG.

The study was reviewed and approved by the Ethics Committee of Sichuan Academy of Medical Sciences & Sichuan Provincial People's Hospital, and conducted in accordance with the Declaration of Helsinki.

### Questionnaire

The most important scale for the assessment of daytime sleepiness is ESS published in 1991 by Murray Johns^28^. It consists of a self-administered questionnaire that investigates the extent of daytime sleepiness specifically and in a very simple manner. There are 8 items in ESS and subjects are asked to rate on a 4-point scale (0–3) his/her chances of dozing in each of 8 different situations that are often encountered in daily life. Arbitrarily, a score of ≧12 has been suggested as being abnormal and an indicator of excessive daytime sleepiness^29^.

### PSG data collection

The gold standard diagnostic method for OSAS is a full-night PSG^30^. The frequency of episodes of apnea and/or hypopnea per hour of sleep, also known as AHI, as well as the lowest observed oxyhemoglobin saturation during sleep is used as the main criteria for severity assessment.

All patients had to have a minimum of 8 h of monitored sleep in the sleep disorder center. They underwent nocturnal PSG monitoring (Philips Alice Version 6, Netherland) using 6 scalp electrodes (C3, C4, F3, F4, O1 and O2 locations), 2 reference electrodes behind the ears (left [A1] and right [A2] mastoid areas), 3 electromyographic electrodes over the submental muscles, 4 electromyographic electrodes over the leg muscles, 2 electrooculographic electrodes, one ground electrode and nasal flow detector. Pulse oximeter was used to obtain nocturnal oximetry recordings. Electrocardiogram was used to obtain rhythm of the heart. The AHI documented the number of apnea-plus-hypopnea incidents every hour during sleep^31^.

### Statistics

Statistical analyses were performed with SPSS 26.0 for mac. The summary of descriptive statistics was presented as mean (with SD) for continuous variables and as frequencies (with percentages) for categorical variables. ANOVA was used for comparison of quantitative data. Univariate and multivariate logistic regression analyses were conducted to identify gender differences of clinical and polysomnographic findings with OSAS. Statistically significant difference was considered if *P* value < 0 0.05^31^.

### Ethics approval and consent to participate

The research was approved by the Ethics Committee of Sichuan Academy of Medical Sciences & Sichuan Provincial People's Hospital. Written informed consent was obtained from all the participants.

### Consent to publish

Written informed consent was obtained from all participants for the publication of any potentially identifiable images or data included in this article.

## Data Availability

The data used and analyzed during the current study are available from the corresponding author on reasonable request.
